# Editorial: Well-being in Asia

**DOI:** 10.3389/fpsyg.2025.1661988

**Published:** 2025-08-06

**Authors:** Martijn J. Burger, Nicholas Tze Ping Pang

**Affiliations:** ^1^Erasmus Happiness Economics Research Organisation, Erasmus University Rotterdam, Rotterdam, Netherlands; ^2^Faculty of Management, Open University of the Netherlands, Heerlen, Netherlands; ^3^C.WAISS and Faculty of Economics, University of Johannesburg, Johannesburg, South Africa; ^4^Faculty of Medicine and Health Sciences, Universiti Malaysia Sabah, Kota Kinabalu, Malaysia; ^5^Unit of Clinical Psychiatry, Department of Clinical Neurosciences/DIMSC, Polytechnic University of Marche, Ancona, Italy

**Keywords:** wellbeing, Asia, China, Taiwan, happiness

Over the past decades, the literature on happiness and wellbeing has been increasing tremendously, a fact corroborated by bibliometric mapping (Veenhoven, 2018; Pang et al., 2023). Yet, a frequent criticism of research on wellbeing and happiness is its heavy reliance on WEIRD populations—those from Western, Educated, Industrialized, Rich, and Developed societies—just like other fields related to psychological science (e.g., Henrich et al., 2010; Hendriks et al., 2019). This raises concerns about whether the field has been disproportionately influenced by Western viewpoints (Lambert et al., 2020; Van Zyl et al., 2024; Steger, 2025), and more importantly whether we truly understand happiness from the perspective of the majority of the population of the world.

For example, comparative studies across countries typically use evaluative indicators like Cantril's Ladder or life satisfaction scales. When emotional balance is assessed, the focus is often on the ratio of high-arousal positive to negative emotions. However, cultural differences in how subjective wellbeing is experienced and valued are significant. For example, East Asian cultures may place greater importance on low-arousal positive states such as tranquility and harmony, rather than on more intense feelings like excitement (Lim, 2016). Additionally, Western ideas of wellbeing often prioritize individual fulfillment, whereby many non-Western cultures view wellbeing as inherently relational or collective (Lambert et al., 2020).

In recent years, an increasing number of studies are beginning to happiness and wellbeing in non-WEIRD contexts, with a precipitous increase in the number of studies from the greater culturally Chinese region (China, Hong Kong, Taiwan/Taipei and Macao) (Easterlin et al., 2021). This Research Topic aims to spotlight wellbeing in Asia in general and the Greater China region in particular, focusing on varieties of hedonic and eudaimonic wellbeing, as well as general and domain-specific wellbeing outcomes such as life satisfaction, meaning in life, psychological wellbeing, and work engagement (see Wijngaards et al., 2022 for a taxonomy).

## In this research topic

Most articles in this Research Topic focus on the wellbeing of younger populations, a topic of pivotal importance as younger populations are experiencing a mental health crisis globally (Blanchflower, 2025).

The study by Wan et al. examines variations in psychological wellbeing across students, reporting that females, only children, first-year students, urban students and students that receive less financial assistance report higher levels of psychological wellbeing. On the same theme, Cui et al. study the relationship between gratitude and academic engagement in Chinese students and find a positive relationship between the two variables that is serially mediated by locus of control and subjective feelings of happiness.

An article by Rudolf presents a cross-national comparison of adolescent life satisfaction, affective wellbeing, and meaning in life. One main finding is that adolescent wellbeing outcomes are not only related to global predictors of subjective wellbeing (such as relationship quality and social economic status), but that also several culture-specific explanations explain the gap in subjective wellbeing across world regions. Most notably, in Confucian East Asia (Greater China, Japan, Korea, Singapore, and Vietnam), low levels of life satisfaction of adolescents are associated with low self-efficacy, low peer wellbeing, as well as with high emotional interdependence compared to other world regions. Moreover, emotional interdependence is more strongly associated with life satisfaction in Confucian East Asia compared to other parts of the world.

The importance of interdependence is also reflected in the study of Han and Yuen, which addresses the role of parental support for life satisfaction and educational hope, defined as a *combination of educational aspirations and goal commitment in education*. The authors find for high school students in Jiangsu (China) that students that receive more intangible support (information, advice, encouragement, praise and care) from their parents are more satisfied with their lives, and this effect is partly mediated by goal commitment.

In a similar vein, Li et al. examine how initial levels of family functioning (including communication, emotional support, parental concern and harmony in the family) affect positive youth development, which is conceptualized as a set of developmental attributes that preadolescents and adolescents need to become contributing members of society. Using four waves of data and latent growth curve modeling, the authors—amongst others—find initial levels of healthy family functioning significantly and positively predicted both Chinese students' initial level and change in positive youth development. The role of the family in adolescents' wellbeing in China is also evident in the study of Zhou et al., who examine meaning of life in a sample of Chinese adolescents. The authors find that adolescents from intact families with higher educated mothers report a higher meaning in life and better mental health outcomes.

Although most studies in the Research Topic focus on adolescents, it is important also to gauge wellbeing at younger ages. Given that it is sometimes difficult to gauge subjective wellbeing of children using traditional scales, Xiang and Choi developed an assessment tool for measuring happiness among Chinese preschoolers. They verified its reliability and validity in a sample of preschoolers aged 3–5 years from kindergartens and childcare centers in the Hangzhou region. Feng et al. on the other focus on the occupational wellbeing of school counselors, who play an important role in safeguarding the mental health of students (Fang et al., 2025). It is of critical importance to understand the main drivers of work-related wellbeing for this occupational group as it is related to work efficacy. The authors find that organizational support, student support, and satisfaction of psychological needs (particularly autonomy and competence) are associated with higher counselors' wellbeing.

Other studies in the Research Topic utilize the China Family Panel Studies and examine the roles of educational inequality and digital technologies for subjective wellbeing respectively. The study by Lin and Liu finds that a higher degree of educational inequality in a region is negatively associated with residents' happiness levels, particularly in lower developed rural areas and central and western regions. The authors also find that the relationship between educational inequality and happiness is mediated by income inequality and lower levels of economic development. Hu et al. report a positive association between the use of digital technologies and happiness, but the association is stronger for women, younger people, primary and college graduates, rural residents, and people with lower incomes. In addition, the authors find that health status, interpersonal relationships, employment situations and income levels mediate the relation between digital technologies and happiness.

The final two studies address the wellbeing of vulnerable populations. Hsu et al. find two distinct subjective wellbeing trajectories among Taiwanese retirees using latent growth mixture models: a group with high-increased wellbeing and a group with a low-declined wellbeing. The first group was characterized by a better health, stronger economic status, greater social participation, more family support, and higher educational attainment, highlighting the importance of different resources during retirement. Finally the study by Zhang and Cao examined relationships between dyadic coping, marital adjustment, and post-traumatic growth in patients with maintenance hemodialysis and their spouses, showing that marital adjustment partially mediates the relationship between dyadic and posttraumatic growth.

## Directions for future research on wellbeing in Asia

Overall, the studies in this Research Topic underscore that wellbeing in Asia, specifically in the Greater China region, cannot be adequately captured by frameworks rooted in Western societies. The studies in this Research Topic on Asian wellbeing underscore that social context plays an important role in shaping wellbeing. Hence, this Research Topic on wellbeing in Asia highlights the limitations of applying a universal model of well-being and points toward the necessity of developing more culturally grounded theories that recognize non-Western values as legitimate pathways to a good life. Perhaps most importantly, this Research Topic challenges Western scholars to change their view of wellbeing and to recognize that the future of wellbeing science lies not in universalizing WEIRD models. Instead, there is a need to pluralize global perspectives and co-construct a more inclusive and representative science of wellbeing.

What directions should future research on wellbeing in Asia take? First, although the majority of studies on Asia currently focus on East Asia (Greater China, Japan, and South Korea), more work is needed on other parts of the continent. In particular, it is important to address the extent to which conceptualizations of wellbeing differ across Asian cultures and how values such as relational harmony, religiousness, and duty affect wellbeing in these societies. WEIRD models emphasize autonomy and self-esteem, but to what extent do these values contribute to wellbeing in Asian societies? Likewise, WEIRD models focus on hedonic wellbeing (Joshanloo and Jarden, 2016), but should eudaimonic wellbeing not be the central focus in studies on Asian wellbeing, particularly given that the Eastern concept of happiness is mostly eudaimonic in nature (Joshanloo, 2014)?

Second, future research should examine how valid existing wellbeing measures are in Asian contexts. For example, it is known that the Cantril ladder often elicits thoughts about power and wealth (Nilsson et al., 2024). However, is this also the case in Asian cultures? In this regard, there is still insufficient understanding of the extent to which wellbeing scales can be compared across cultures. Without proper measurement tools, wellbeing levels are easily misinterpreted. In addition, it should be examined to what extent indigenous constructs better capture culturally specific dimensions of wellbeing, such as amae in Japanese (Niiya et al., 2006) and Ananda in India (e.g., Nagar, 2018). Should we develop not only universal tools but also more culturally sensitive measurement scales?

Third, it is important to study the developing Asian context and how it drives wellbeing in the region. Particular attention should be given to geopolitical tensions across the Asian continent, as well as societal developments such as rapid urbanization and increasing educational pressure in some Asian countries. It would also be valuable to examine the roles of informal economies, kin networks, and communal safety nets in influencing wellbeing. Finally, governments appear to play a substantial role in shaping daily life in many Asian societies. How do national frameworks such as Gross National Happiness in Bhutan or Vision 2030 in Saudi Arabia shape wellbeing in these countries? These are all important questions that future research could address.
